# Causal relationships between blood metabolites and diabetic retinopathy: a two-sample Mendelian randomization study

**DOI:** 10.3389/fendo.2024.1383035

**Published:** 2024-05-01

**Authors:** Chongchao Yang, Yan Ma, Mudi Yao, Qin Jiang, Jinsong Xue

**Affiliations:** ^1^ The Affiliated Eye Hospital, Nanjing Medical University, Nanjing, Jiangsu, China; ^2^ The Fourth School of Clinical Medicine, Nanjing Medical University, Nanjing, Jiangsu, China; ^3^ Department of Ophthalmology, The First People’s Hospital, Shanghai, China

**Keywords:** diabetic retinopathy, blood metabolites, Mendelian randomization, metabolic pathway analysis, meta-analysis

## Abstract

**Background:**

Diabetic retinopathy (DR) is a microvascular complication of diabetes, severely affecting patients’ vision and even leading to blindness. The development of DR is influenced by metabolic disturbance and genetic factors, including gene polymorphisms. The research aimed to uncover the causal relationships between blood metabolites and DR.

**Methods:**

The two-sample mendelian randomization (MR) analysis was employed to estimate the causality of blood metabolites on DR. The genetic variables for exposure were obtained from the genome-wide association study (GWAS) dataset of 486 blood metabolites, while the genetic predictors for outcomes including all-stage DR (All DR), non-proliferative DR (NPDR) and proliferative DR (PDR) were derived from the FinnGen database. The primary analysis employed inverse variance weighted (IVW) method, and supplementary analyses were performed using MR-Egger, weighted median (WM), simple mode and weighted mode methods. Additionally, MR-Egger intercept test, Cochran’s *Q* test, and leave-one-out analysis were also conducted to guarantee the accuracy and robustness of the results. Subsequently, we replicated the MR analysis using three additional datasets from the FinnGen database and conducted a meta-analysis to determine blood metabolites associated with DR. Finally, reverse MR analysis and metabolic pathway analysis were performed.

**Results:**

The study identified 13 blood metabolites associated with All DR, 9 blood metabolites associated with NPDR and 12 blood metabolites associated with PDR. In summary, a total of 21 blood metabolites were identified as having potential causal relationships with DR. Additionally, we identified 4 metabolic pathways that are related to DR.

**Conclusion:**

The research revealed a number of blood metabolites and metabolic pathways that are causally associated with DR, which holds significant importance for screening and prevention of DR. However, it is noteworthy that these causal relationships should be validated in larger cohorts and experiments.

## Introduction

Diabetic retinopathy (DR) stands as the predominant and severe ocular complication arising from diabetes mellitus. It ranks as the foremost contributor to irreversible yet preventable vision impairment among the working-age adult population (1). With the rapid increase in the incidence of diabetes worldwide, the number of people with DR is expected to rise to about 161 million by 2045 (2). According to the classification of Airlie House, DR is classified into non-proliferative DR (NPDR) and proliferative DR (PDR) (3). NPDR is the early manifestation of DR, characterized primarily by retinal microaneurysms, hemorrhages, hard exudates, and cotton lint spots (3). PDR is the advanced stage of DR, characterized by retinal neovascularization (3). The pathogenesis of DR is complex and includes multiple contributing factors such as oxidative stress, inflammation, angiogenesis, intestinal flora dysregulation, and neurodegeneration (4). Common risk factors for DR include diabetes course (5), elevated blood glucose levels (6), high lipid levels (7), and hypertension (8). Despite the extensive research conducted on DR, the mechanisms and risk factors for DR are still not fully understood.

Presently, an expanding body of research suggests a close correlation between metabolic disturbance and DR (9, 10). Furthermore, metabolomics is a powerful tool that greatly aids in identifying differential metabolites in DR (10, 11). These metabolites are often potential biomarkers and targets for the disease and can be utilized for screening, prediction, and treatment of DR (12, 13). However, exploring causal relationships between metabolites and DR is challenging due to limited sample sizes and confounding factors. Randomized controlled trials (RCTs) are generally recognized as the best evidence for epidemiological studies, but they require substantial resources and time, and ethical concerns may sometimes make them impractical. As an alternative, Mendelian randomization (MR) study explores the causality of exposure on outcome by employing single nucleotide polymorphisms (SNPs) as instrumental variables (IVs) (14). Recently, genome-wide association study (GWAS) has updated metabolic phenotypes that created a genetically determined metabolites (GDMs) atlas (15). There is no MR study investigating the causal relationships between circulating metabolites and DR. Our research aims to identify blood metabolites associated with DR and provide new perspectives on its biological processes.

## Materials and methods

### Study design

**Figure 1:**
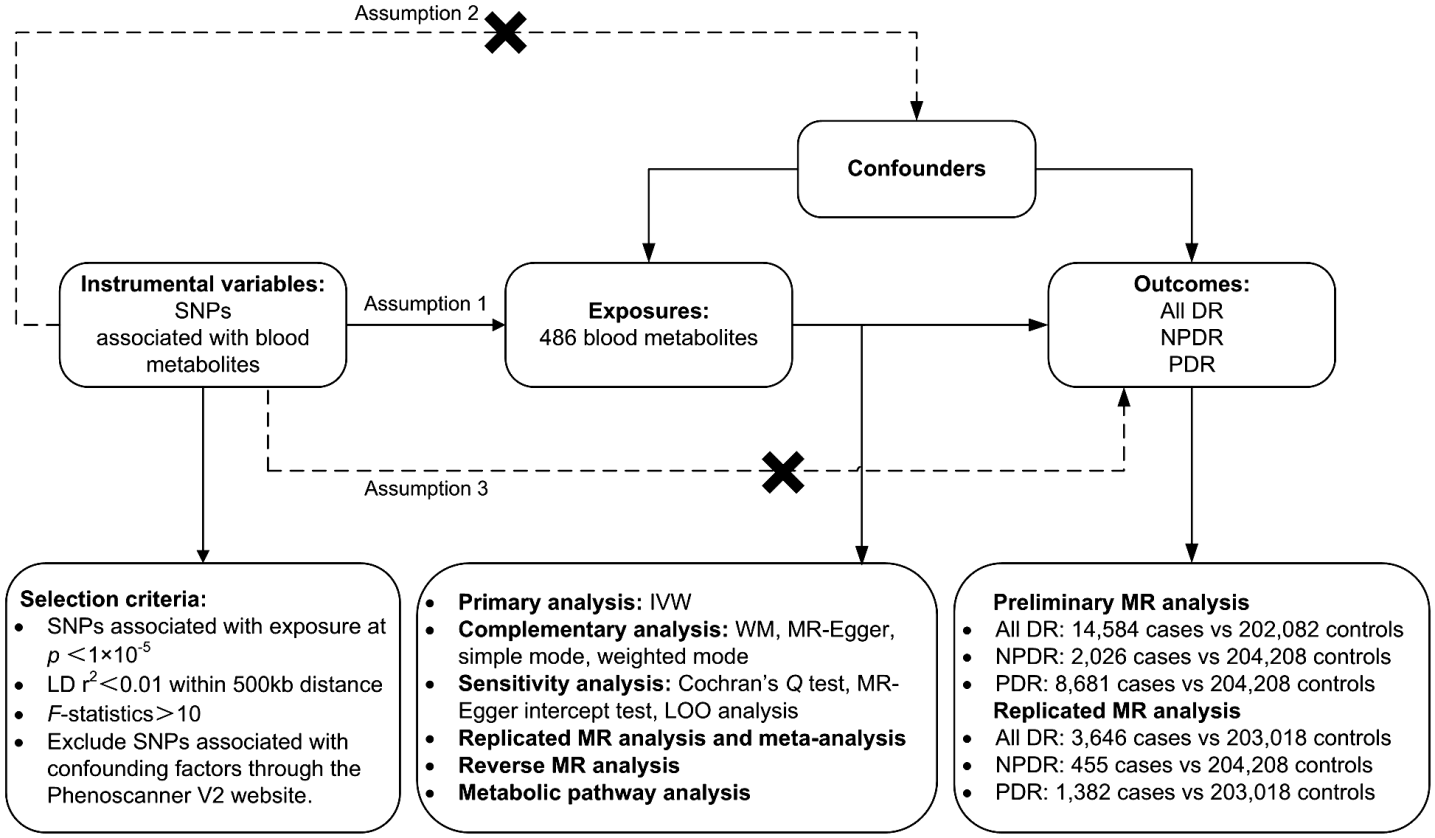
Study design, datasets, assumptions of the Mendelian randomization (MR) study of the associations between 486 blood metabolites and diabetic retinopathy. nSNPs, number of single nucleotide polymorphisms; LD, linkage-disequilibrium.

A two-sample MR analysis was utilized to assess the causality of human circulating metabolites on the risk of DR. Summary data for the exposures (486 blood metabolites) and outcomes (DR) were both sourced from GWAS. Ensuring the effectiveness of MR analysis requires satisfying three assumptions: (1) There should be a close connection between genetic variations and exposure; (2) the genetic variations ought to be independent of confounders related to exposure and outcome; (3) the genetic variations ought to be unrelated to outcome and only affect outcome through exposure. To prevent sample duplication, the hereditary information of metabolites and DR was derived from distinct datasets (**Figure 1**). The report followed the STROBE-MR statement (16).

### Data sources for blood metabolites

Genetic variations of 486 blood metabolites were obtained through comprehensive genetic scanning and metabolic analyses performed by Shin et al. (15). These publicly available data were derived from the GWAS Catalog (http://metabolomics.helmholtz-muenchen.de/gwas/). The dataset identifies about 2.1 million SNPs from 7,824 adults across two European cohorts (TwinsUK and KORA cohorts). According to the Kyoto Encyclopedia of Genes and Genomes (KEGG) database (17), 309 known metabolites are categorized into 8 pathways: amino acids, lipids, cofactors and vitamins, carbohydrates, nucleotides, energy, peptides and xenobiotics. The chemical properties of another 177 unknown metabolites have yet to been determined (**Supplementary Table 1**).

### Data sources for DR

Based on different stages of DR, outcomes are categorized into all-stage DR (All DR), NPDR, and PDR. The summary dataset of genetic variants related to DR was sourced from FinnGen (https://r5.finngen.fi/). The GWAS IDs for the outcomes are as follows: All DR (finn-b-DM_RETINOPATHY); NPDR (finn-b-DM_BCKGRND_RETINA); PDR (finn-b-DM_RETINA_PROLIF). The characteristics of these summary datasets are presented in **Table 1**.

**Table 1:** Characteristics of the summary datasets for diabetic retinopathy in preliminary MR Analysis.

DR stage	Population	Sample size	Case	Control	nSNPs
All DR	European	216,666	14,584	202,082	16,380,459
NPDR	European	206,234	2,026	204,208	16,380,446
PDR	European	212,889	8,681	204,208	16,380,460

nSNPs, number of single nucleotide polymorphisms.

### IVs selection

We established several criteria for screening IVs associated with blood metabolites. Firstly, considering the relatively small number of SNPs associated with metabolites, we lowered the significance threshold to *p <*1×10^–5^ to ensure a comprehensive conclusion (18). Secondly, r^2^ < 0.01 within 500-kilobase (kb) distance was set as linkage-disequilibrium (LD) threshold (19, 20) and the SNP of the moderate frequency palindrome structure was excluded. Thirdly, we employed *F*-statistic to evaluate the power of IVs. In general, IVs with *F*-statistic > 10 were used for subsequent MR analysis (19). The above criteria have been applied in previous literatures (18, 20). In addition, we examined these SNPs on the Phenoscanner V2 website (http://www.phenoscanner.medschl.cam.ac.uk/) to evaluate whether they are related to common confounders for DR, such as Type 1 Diabetes (5), Type 2 Diabetes (T2D) (21), Diabetic nephropathy (DN) (22), HbA1c (6), blood pressure (8), fasting glucose (6), fasting insulin (23), Total Cholesterol (7), Body Mass Index (24) and smoking (25). SNPs related to the above confounders (*p* < 1×10^–5^) were excluded to satisfy the MR assumptions.

### MR analysis and sensitivity analysis

We primarily evaluated the causality of 486 metabolites on DR through random‐effects inverse variance weighted (IVW) method, which yields the most reliable estimation results when all chosen SNPs serve as valid IVs (14). If IVs challenge the MR assumptions, the results could be inaccurate. Consequently, we conducted the subsequent sensitivity analyses to ensure the reliability of our results: (1) Cochran’s *Q* test was employed to assess heterogeneity among SNPs; (2) MR-Egger intercept was calculated to detect horizontal pleiotropy; (3) Supplementary analyses including MR-Egger, weighted median (WM), weighted mode and simple mode were used to guarantee the stability and dependability of the conclusion; (4) Leave‐one‐out (LOO) method was employed to assess whether the results were greatly influenced by individual SNP. The analyses were carried out utilizing the TwoSampleMR and MRPRESSO packages in the R software (version 4.3.2). The threshold of significance was set as *P* < 0.05.

### Replicated MR analysis and meta‐analysis

To ensure the reliability and stability of the preliminary MR analysis results, we replicated the MR analysis using three additional DR datasets following the above steps. The summary data for DR in the repetitive analysis were also obtained from the FinnGen database, and the GWAS IDs for these datasets are as follows: All DR (finn-b-H7_RETINOPATHYDIAB), NPDR (finn-b-DM_BCKGRND_RETINA_NONPROLIF) and PDR (finn-b-H7_RETINOPATHYDIAB_PROLIF). The characteristics of these summary datasets are presented in **Table 2**. Through a meta-analysis of two datasets, we conclusively identified blood metabolites causally linked to DR. The Review Manager (version 5.4) was used for the meta-analysis with random-effects IVW model.

**Table 2:** Characteristics of the summary datasets for diabetic retinopathy in replicated MR Analysis.

DR stage	Population	Sample size	Case	Control	nSNPs
All DR	European	206,664	3,646	203,018	16,380,430
NPDR	European	204,663	455	204,208	16,380,421
PDR	European	204,400	1,382	203,018	16,380,401

nSNPs, number of single nucleotide polymorphisms.

### Reverse MR analysis

To further explore the causality of DR on circulating metabolites, we performed a reverse MR analysis using DR as the exposure and the identified metabolites as the outcomes. *p* < 5×10^–8^ and r^2^ < 0.001 within 10,000-kilobase (kb) distance were set as clumping threshold to extract DR-related IVs.

### Metabolic pathway analysis

To clarify the roles of circulating metabolites in the pathogenesis of DR, we conducted Metaboanalyst 6.0 (https://www.Metaboanalyst.ca/) for metabolic pathway analysis. Metabolite names were standardized to HMDB IDs based on the HMDB database (https://hmdb.ca/) for metabolic pathway analysis. *P* < 0.05 was selected as the significance threshold.

## Results

### IVs selection

The filtered IVs comprised SNPs ranging from 2 to 481 (Glutamate had the fewest IVs, with 2 SNPs, while 2-methoxyacetaminophen sulfate had the most IVs, with 481 SNPs). These SNPs associated with metabolites exhibited *F* statistics greater than 10 (**Supplementary Tables 2, 3, 4**), and they were not related to above confounding factors after examination by the Phenoscanner V2 website.

### Causal effect of metabolites on DR

**Figure 2:**
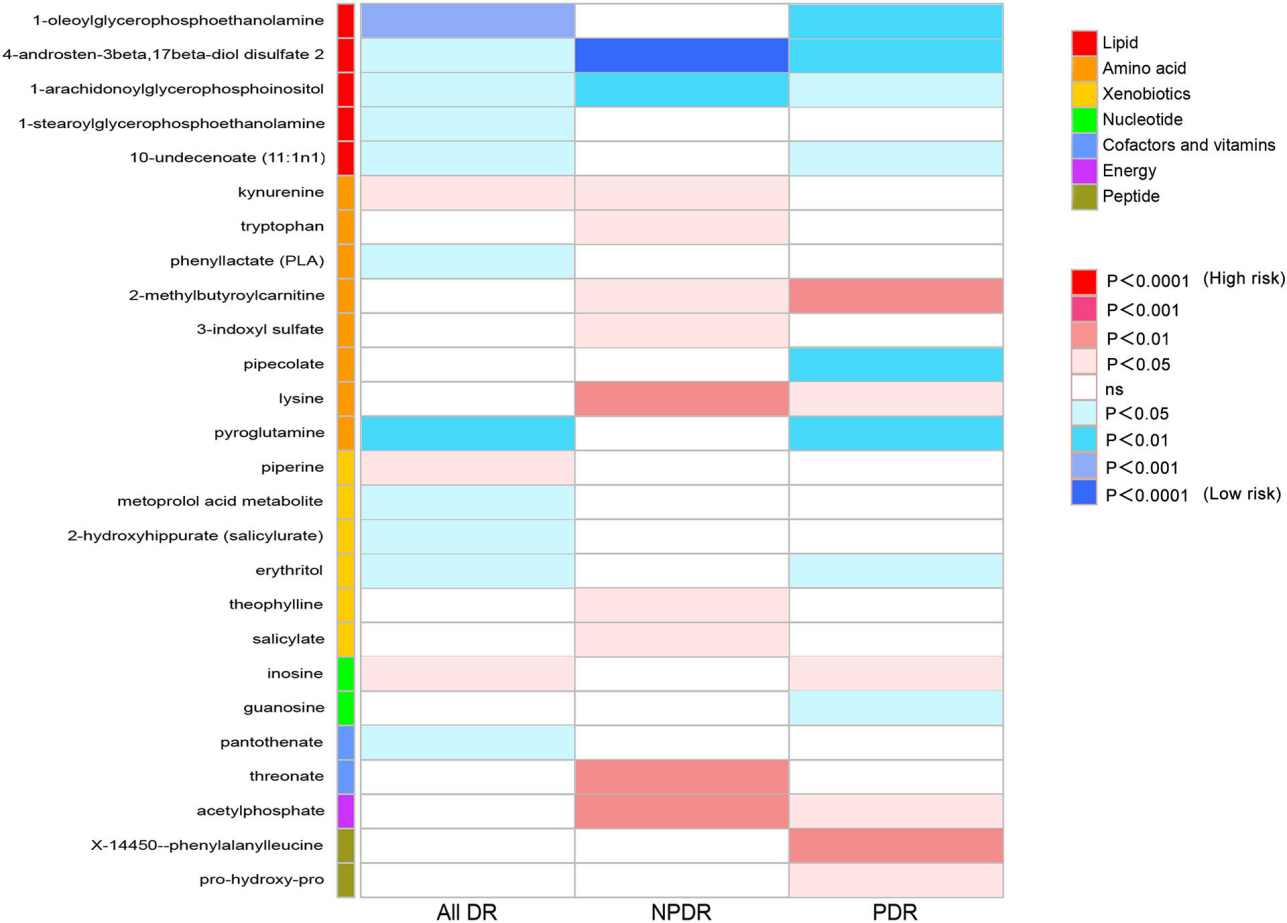
Heat map of causal associations between blood metabolites and diabetic retinopathy (derived from IVW analysis, *p* < 0.05). IVW, inverse-variance weighted.

To provide a better understanding of metabolic changes, 177 unknown metabolites were excluded, while 309 metabolites with known structure and function were included. We estimated the causality of 309 blood metabolites on DR and found 39 significant associations, corresponding to 26 different metabolites, which included eight metabolites in the amino acid pathways, five in the lipid metabolism pathways, six in the xenobiotic pathways, two in the nucleotide pathways, two in the cofactors and vitamins pathways, one in the energy pathways, two in the peptide pathways (**Figure 2**). The complete results were provided in **Supplementary Table 5**. The causality of 486 metabolites on All DR, NPDR and PDR were shown in **Supplementary Tables 6, 7, 8** respectively.

### Causal effects of metabolites on All DR

**Figure 3:**
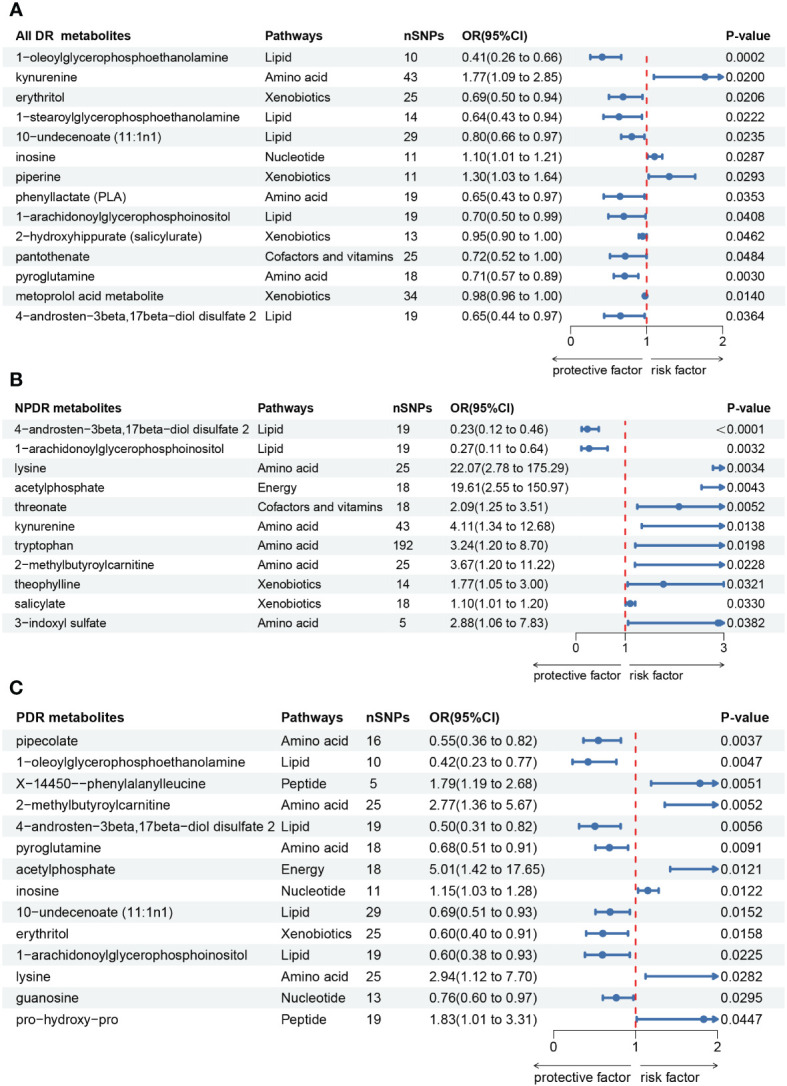
Forest plot of Mendelian randomization (MR) associations of blood metabolites on the risk of diabetic retinopathy (derived from IVW analysis, *p* < 0.05). **(A)** all-stage diabetic retinopathy (All DR); **(B)** non-proliferative diabetic retinopathy (NPDR); **(C)** proliferative diabetic retinopathy (PDR). IVW, inverse-variance weighted; 95%CI, 95% confidence interval; OR, odds ratio; nSNPs, number of single nucleotide polymorphisms.

Firstly, we identified 14 blood metabolites associated with All DR, as shown in **Figure 3A**. They are as follows: 1-oleoylglycerophosphoethanolamine [OR = 0.41, 95%CI = (0.26, 0.66), *p* = 0.0002]; kynurenine [OR = 1.77, 95%CI = (1.09, 2.85), *p* = 0.0200]; erythritol [OR = 0.69, 95%CI = (0.50, 0.94), *p* = 0.0206]; 1-stearoylglycerophosphoethanolamine [OR = 0.64, 95%CI = (0.43, 0.94), *p* = 0.0222]; 10-undecenoate (11:1n1) [OR = 0.80, 95%CI = (0.66, 0.97), *p* = 0.0235]; inosine [OR = 1.10, 95%CI = (1.01, 1.21), *p* = 0.0287]; piperine [OR = 1.30, 95%CI = (1.03, 1.64), *p* = 0.0293]; phenyllactate (PLA) [OR = 0.65, 95%CI = (0.43, 0.97), *p* = 0.0353]; 1-arachidonoylglycerophosphoinositol [OR = 0.70, 95%CI = (0.50, 0.99), *p* = 0.0408]; 2-hydroxyhippurate (salicylurate) [OR = 0.95, 95%CI = (0.90, 1.00), *p* = 0.0462]; pantothenate [OR = 0.72, 95%CI = (0.52, 1.00), *p* = 0.0484]; pyroglutamine [OR = 0.71, 95%CI = (0.57, 0.89), *p* = 0.0030]; metoprolol acid metabolite [OR = 0.98, 95%CI = (0.96, 1.00), *p* = 0.0140]; 4-androsten-3beta,17beta-diol disulfate 2 [OR = 0.65, 95%CI = (0.44, 0.97), *p* = 0.0364].

### Causal effects of metabolites on NPDR

Secondly, we identified 11 blood metabolites associated with NPDR, as shown in **Figure 3B**. They are as follows: 4-androsten-3beta,17beta-diol disulfate 2 [OR = 0.23, 95%CI = (0.12, 0.46), *p* < 0.0001]; 1-arachidonoylglycerophosphoinositol [OR = 0.27, 95%CI = (0.11, 0.64), *p* = 0.0032]; lysine [OR = 22.07, 95%CI = (2.78, 175.29), *p* = 0.0034]; acetylphosphate [OR = 19.61, 95%CI = (2.55, 150.97), *p* = 0.0043]; threonate [OR = 2.09, 95%CI = (1.25, 3.51), *p* = 0.0052]; kynurenine [OR = 4.11, 95%CI = (1.34, 12.68), *p* = 0.0138]; tryptophan [OR = 3.24, 95%CI = (1.20, 8.70), *p* = 0.0198]; 2-methylbutyroylcarnitine [OR = 3.67, 95%CI = (1.20, 11.22), *p* = 0.0228]; theophylline [OR = 1.77, 95%CI = (1.05, 3.00), *p* = 0.0321]; salicylate [OR = 1.10, 95%CI = (1.01, 1.20), *p* = 0.0330]; 3-indoxyl sulfate [OR = 2.88, 95%CI = (1.06, 7.83), *p* = 0.0382].

### Causal effects of metabolites on PDR

Thirdly, we identified 14 blood metabolites associated with PDR, as shown in **Figure 3C**. They are as follows: pipecolate [OR = 0.55, 95%CI = (0.36, 0.82), *p* = 0.0037]; 1-oleoylglycerophosphoethanolamine [OR = 0.42, 95%CI = (0.23, 0.77), *p* = 0.0047]; X-14450–phenylalanylleucine [OR = 1.79, 95%CI = (1.19, 2.68), *p* = 0.0051]; 2-methylbutyroylcarnitine [OR = 2.77, 95%CI = (1.36, 5.67), *p* = 0.0052]; 4-androsten-3beta,17beta-diol disulfate 2 [OR = 0.50, 95%CI = (0.31, 0.82), *p* = 0.0056]; pyroglutamine [OR = 0.68, 95%CI = (0.51, 0.91), *p* = 0.0091]; acetylphosphate [OR = 5.01, 95%CI = (1.42, 17.65), *p* = 0.0121]; inosine [OR = 1.15, 95%CI = (1.03, 1.28), *p* = 0.0122]; 10-undecenoate (11:1n1) [OR = 0.69, 95%CI = (0.51, 0.93), *p* = 0.0152]; erythritol [OR = 0.60, 95%CI = (0.40, 0.91), *p* = 0.0158]; 1-arachidonoylglycerophosphoinositol [OR = 0.60, 95%CI = (0.38, 0.93), *p* = 0.0225]; lysine [OR = 2.94, 95%CI = (1.12, 7.70), *p* = 0.0282]; guanosine [OR = 0.76, 95%CI = (0.60, 0.97), *p* = 0.0295]; pro-hydroxy-pro [OR = 1.83, 95%CI = (1.01, 3.31), *p* = 0.0447].

### Sensitive analysis

**Figure 4:**
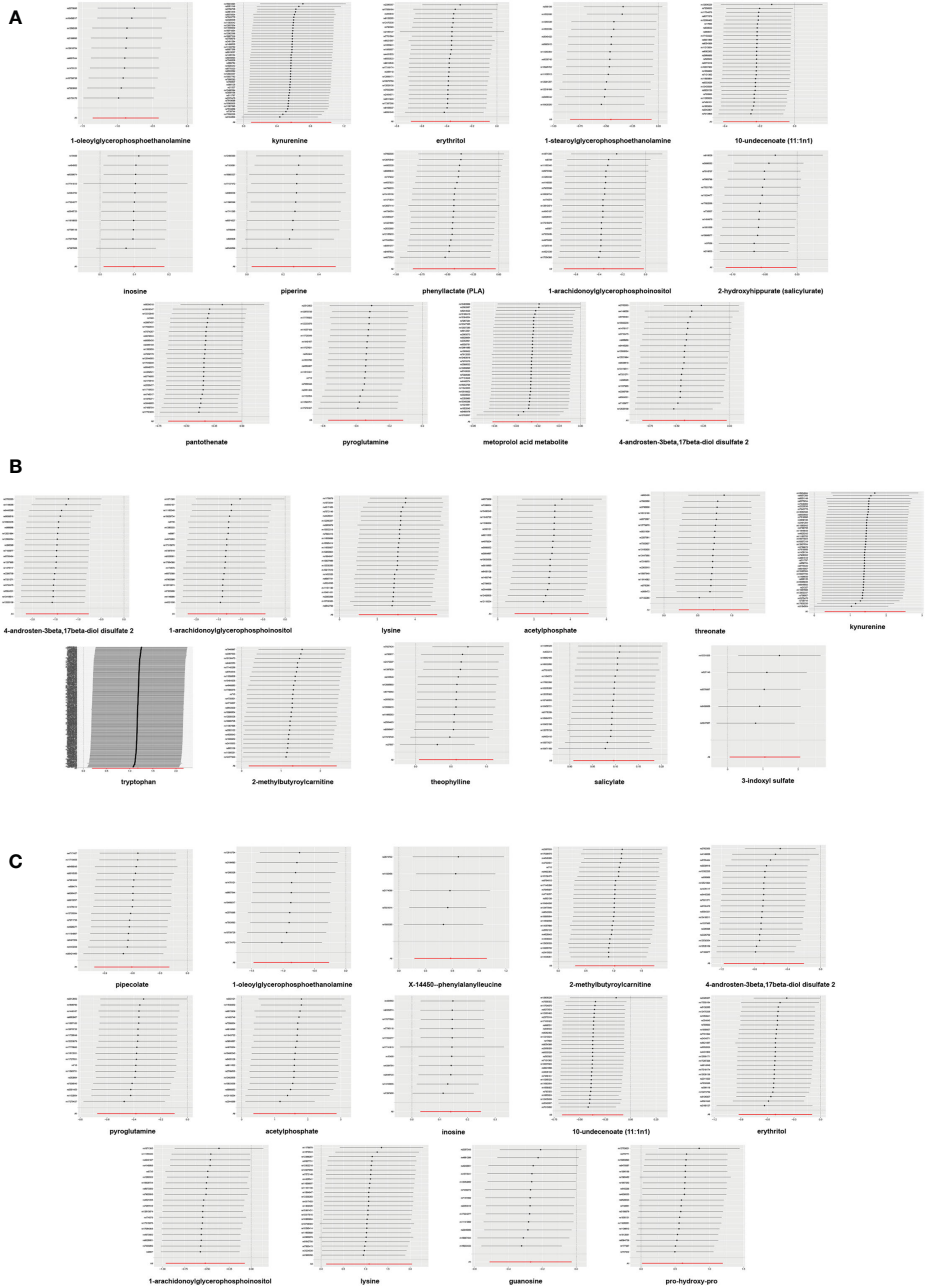
Leave-one-out analysis of Mendelian randomization (MR) analyses between blood metabolites and diabetic retinopathy. **(A)** all-stage diabetic retinopathy (All DR); **(B)** non-proliferative diabetic retinopathy (NPDR); **(C)** proliferative diabetic retinopathy (PDR).

**Figure 5:**
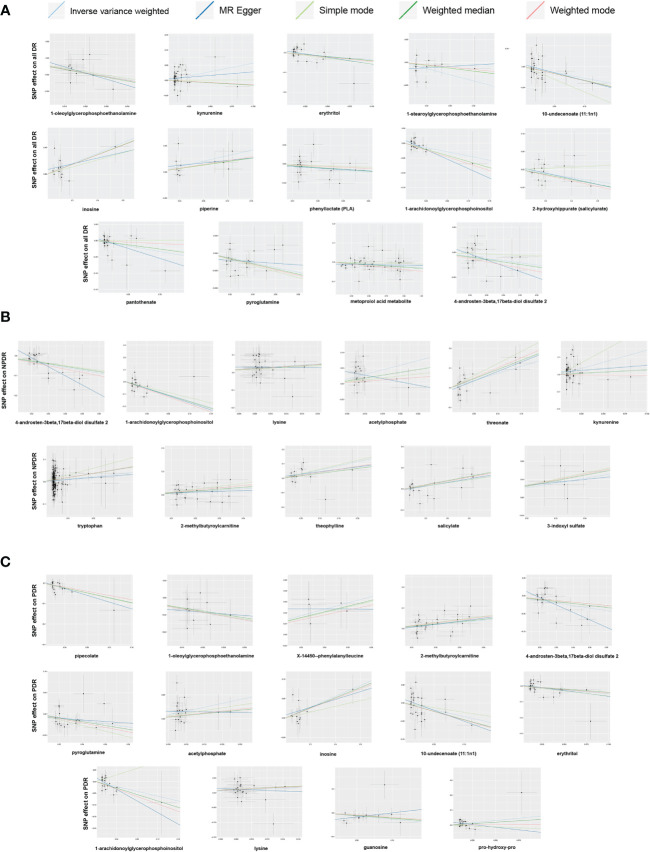
Scatter plots of Mendelian randomization (MR) analyses between blood metabolites and diabetic retinopathy. **(A)** all-stage diabetic retinopathy (All DR); **(B)** non-proliferative diabetic retinopathy (NPDR); **(C)** proliferative diabetic retinopathy (PDR).

**Figure 6:**
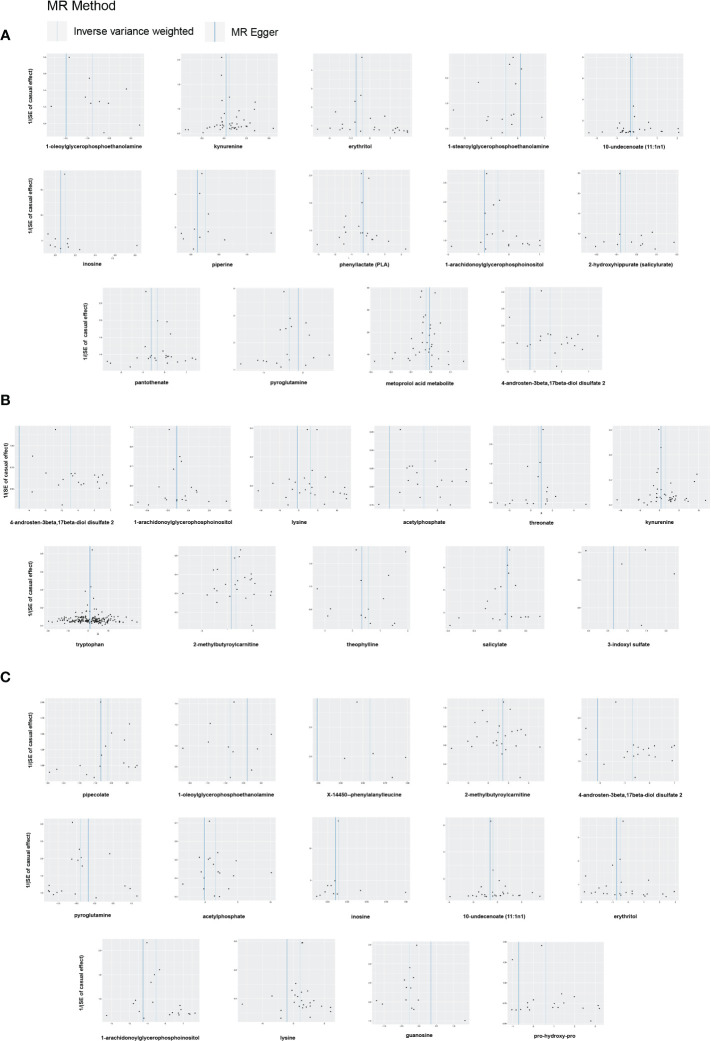
Funnel plots of Mendelian randomization (MR) analyses between blood metabolites and diabetic retinopathy. **(A)** all-stage diabetic retinopathy (All DR); **(B)** non-proliferative diabetic retinopathy (NPDR); **(C)** proliferative diabetic retinopathy (PDR).

In sensitivity analysis, the results of complementary analyses including MR-Egger, weighted median (WM), simple mode and weighted mode are shown in **Supplementary Table 5**. We did not observe horizontal pleiotropy in 39 significant associations by MR-Egger intercepts (**Supplementary Table 5**). However, we found significant heterogeneity for some metabolites by Cochrane’s *Q*-test (**Supplementary Table 5**). They are as follows: All DR: kynurenine (IVW: *Q* = 72.71, *p* = 0.0023; MR-Egger: *Q* = 71.76, *p* = 0.0021) and 4-androsten-3beta,17beta-diol disulfate 2 (IVW: *Q* = 39.20, *p* = 0.0027; MR-Egger: *Q* = 36.59, *p* = 0.0038); NPDR: kynurenine (IVW: *Q* = 61.55, *p* = 0.0261; MR-Egger: *Q* = 60.21, *p* = 0.0268); PDR: 2-methylbutyroylcarnitine (IVW: *Q* = 40.90 *p* = 0.0171; MR-Egger: *Q* = 40.61, *p* = 0.0131); 4-androsten-3beta,17beta-diol disulfate 2 (IVW: *Q* = 35.71, *p* = 0.0077; MR-Egger: *Q* = 30.03, *p* = 0.0261); 10-undecenoate (11:1n1) (IVW: *Q* = 45.60, *p* = 0.0192; MR-Egger: *Q* = 41.72, *p* = 0.0351). To reduce the impact of heterogeneity on the results, we used the random-effects IVW method to calculate causal effects of metabolites mentioned above on DR. Finally, the LOO analysis showed that the overall effect of the metabolites was not strongly influenced by any single SNP (**Figure 4**). In addition, the scatter plots and funnel plots of MR analyses for the identified blood metabolites are shown in **Figures 5**, **6**.

### Replicated MR analysis and meta‐analysis

**Figure 7:**
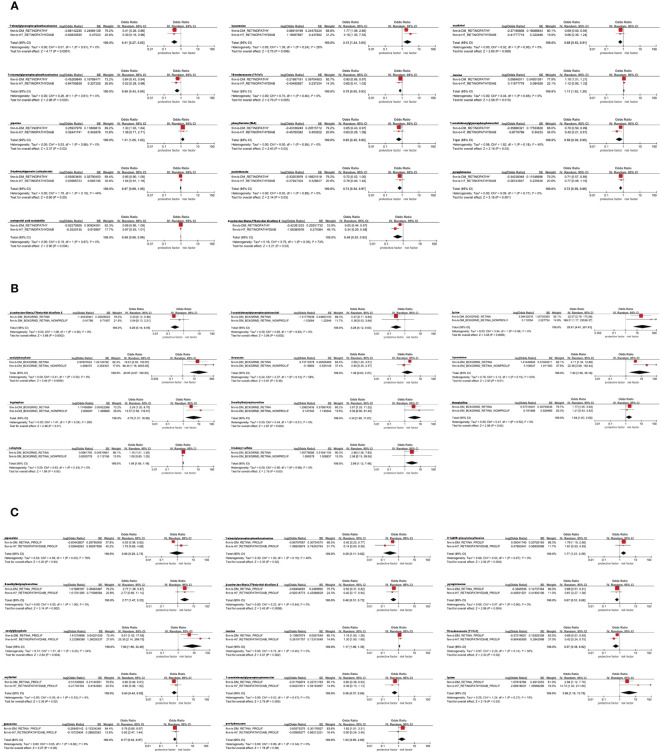
Meta-analysis of the causal associations between blood metabolites and diabetic retinopathy. **(A)** all-stage diabetic retinopathy (All DR); **(B)** non-proliferative diabetic retinopathy (NPDR); **(C)** proliferative diabetic retinopathy (PDR). 95% CI, 95% confidence interval; OR, odds ratio.

To improve the credibility of the results, we performed a replicated MR analysis using three additional GWAS datasets for DR. As anticipated, we discovered the candidate metabolites with trends analogous to those in the preliminary MR analysis, and without horizontal pleiotropy (**Supplementary Table 9**). In addition, the replicated MR results for 486 metabolites are presented in **Supplementary Tables 10, 11, 12** respectively. Through a meta-analysis of the results from two MR analyses, we ultimately identified 34 significant correlations involving 21 blood metabolites, among which 13 were associated with All DR, 9 with NPDR, and 12 with PDR (**Figure 7**). Pipecolate, 2-hydroxyhippurate (salicylurate), salicylate, threonate and pro-hydroxy-pro were excluded due to non-significant results in the meta-analysis.

### Reverse MR analysis

**Figure 8:**
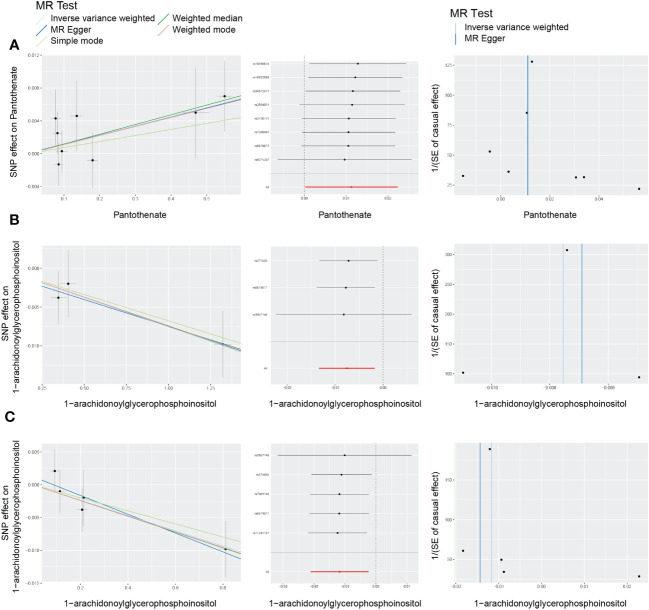
Scatter plots, funnel plots and leave-one-out (LOO) analysis of reverse Mendelian randomization (MR) analyses between blood metabolites and diabetic retinopathy. **(A)** all-stage diabetic retinopathy (All DR); **(B)** non-proliferative diabetic retinopathy (NPDR); **(C)** proliferative diabetic retinopathy (PDR).

Among the 21 ultimately identified metabolites, we found a reverse causal relationship between pantothenate and All DR [OR = 1.011, 95%CI = (1.000, 1.023), *p* = 0.0466], while 1-arachidonoylglycerophosphoinositol exhibited reverse causal effects with both NPDR [OR = 0.992, 95%CI = (0.987, 0.998), *p* = 0.0109] and PDR [OR = 0.988, 95%CI = (0.979, 0.998), *p* = 0.0143]. The results of reverse MR analysis and sensitivity analysis are shown in **Supplementary Table 13**. In addition, the scatter plots, funnel plots and LOO analysis of reverse MR analyses for the identified blood metabolites are shown in **Figure 8**.

### Metabolic pathway analysis

**Figure 9:**
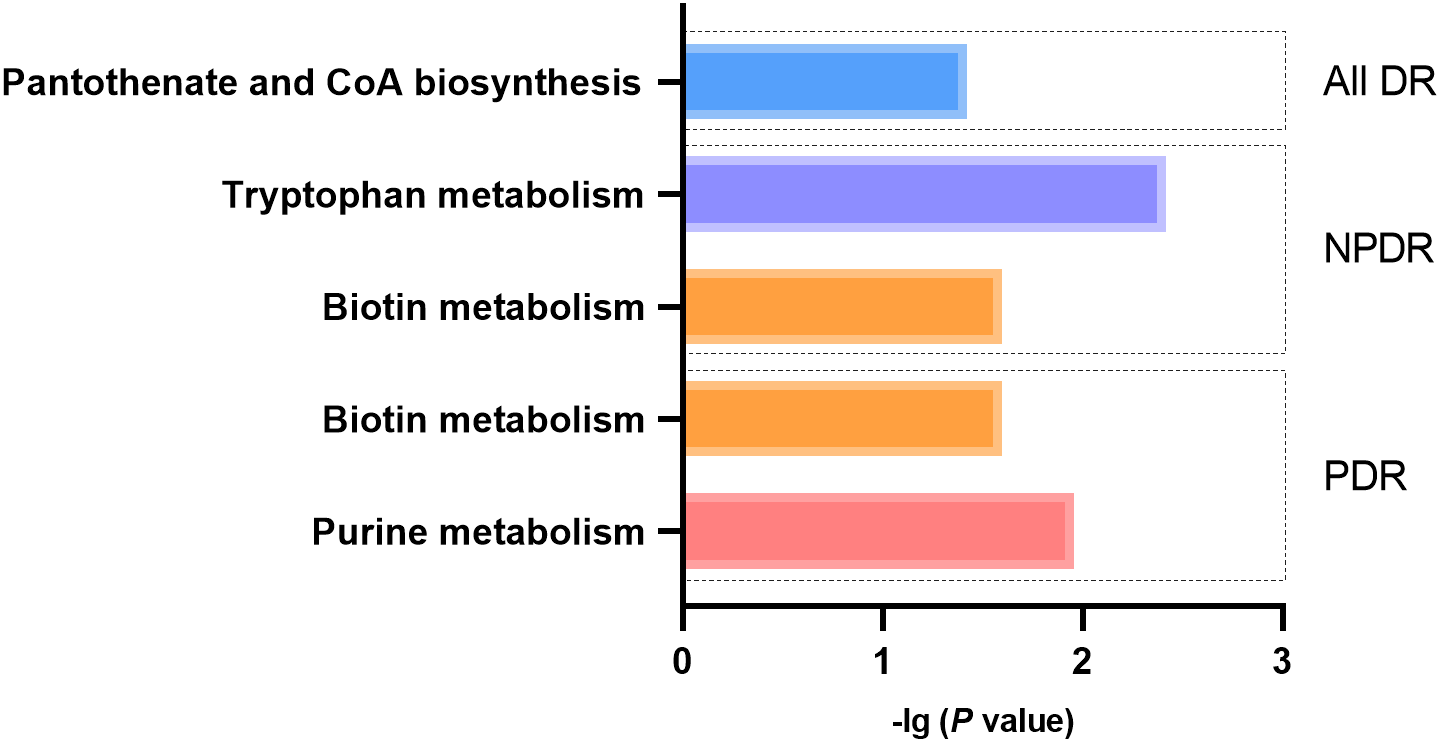
Metabolic pathways with significant enrichment of blood metabolites.

A total of 5 significant associations corresponding to 4 metabolic pathways were identified through metabolic pathway analysis (**Figure 9**). “Pantothenate and CoA biosynthesis” (*p* = 0.038) may be involved in the biological process of All DR. “Tryptophan metabolism” (*p* = 0.004) and “Biotin metabolism” (*p* = 0.025) may be involved in the biological process of NPDR. “Purine metabolism” (*p* = 0.011) and “Biotin metabolism” (*p* = 0.025) may be involved in the biological process of PDR (**Supplementary Table 14**).

## Discussion

Among the 486 blood metabolites, we ultimately identified 13 metabolites associated with All DR, 9 with NPDR, and 12 with PDR, totaling 21 metabolites associated with DR. These findings have significant implications for future research in identifying novel biomarkers and targets for DR, and may inspire new preventive and therapeutic strategies.

Diabetes is widely recognized as a significant global public health issue, leading to microvascular complications such as DN and DR (21, 26). Approximately one-third of diabetes patients develop DR (21). Recent studies revealed that the pathological processes of DR were associated with long-term metabolic disturbances (9, 27, 28). With the advancement of metabolomics, metabolites and metabolic pathways associated with DR are continuously being discovered (10, 27, 29). These crucial metabolites are often considered potential biomarkers for disease prediction. In human studies, various biofluids such as circulating blood (serum and plasma), aqueous humor, vitreous body are used for the detection of metabolites. Due to its ease of acquisition and low invasiveness, circulating blood is the most commonly used sample. It offers a comprehensive profile of metabolic characteristics that can help identify potential biomarkers of DR.

We found causal relationships between seven metabolites in the amino acid metabolic pathway and DR. Amino acids participate in energy metabolism and regulate various metabolic pathways through gluconeogenesis (30). Previous studies have highlighted the significant role of amino acids in DR (30–32). Our findings specifically focused on the roles of tryptophan (TRP) and kynurenines (KYN) in DR. TRP serves as a primary origin for a range of bioactive molecules, including KYN, 5-hydroxytryptamine, melatonin, niacin and indoles (33). The TRP–KYN pathway constitutes the principal route for TRP conversion in central and peripheral tissues (33). The rate-limiting enzymes responsible for the initial step of the TRP-KYN pathway is indoleamine 2,3-dioxygenases (IDO), which is associated with immune response and inflammation (34). Previous research suggested that patients with DR exhibited higher level of retinal IDO compared to non-diabetic patients, and the loss of IDO has been shown to inhibit capillary degeneration in diabetic mice (35). Praveen et al. (36) observed a significant elevation in KYN levels and IDO mRNA levels in the serum of DR patients compared to healthy individuals, with a more pronounced increase in PDR patients. Meanwhile, multiple metabolomics analyses of plasma/serum from DR patients have also identified TRP and KYN as potential biomarkers of DR (10, 37). Indoxyl sulfate, a uremic toxin produced by bacterial decomposition of intestinal amino acids, especially TRP, has been reported to be involved in the metabolic disorder of DN (38). Our research suggested that 3-indoxyl sulfate may serve as a potential risk factor for DR, although further confirmation is required through experiments. Pyroglutamine, a cyclic derivative of glutamine, has been reported to be associated with kidney function and T2D (39). Our study identified pyroglutamine as a potential protective factor for DR.

Lysine primarily undergoes metabolic processes via the saccharopine pathway, with a smaller fraction proceeding through the pipecolic acid pathway (40). Our research identified lysine as a potential risk factor for DR, while pipecolate (pipecolic acid) is considered a potential protective factor against DR in the preliminary MR analysis. A metabolomic analysis observed increased levels of lysine in the vitreous bodies of DR patients compared to the normal population (41). Vidhya et al. (42) also reported a significant elevation of lysine in the vitreous bodies of PDR patients. *In vivo* experiment, lysine was found to promote the differentiation of retinal pericytes into adipocytes, exerting a protective effect on PDR (42), which contradicts with our research finding. As for pipecolic acid, Luo et al. (12) found that the serum levels of pipecolic acid were lower compared to those in healthy population, and it exhibited a negative correlation with blood glucose and glycated hemoglobin. Wang et al. (43) also observed reduced pipecolic acid in plasma and vitreous body of PDR patients through metabolomic analysis. *In vivo* experiment, pipecolic acid was found to alleviate ferroptosis in DR by inhibiting the GPX4-YAP signaling pathway, thereby preventing the progression of DR (12). Although the meta-analysis result for pipecolate was not significant, it may have a certain potential effect in the development of DR.

The disruption of lipid metabolism is generally recognized to is connected with the onset and development of DR (44). The study revealed that the lipid metabolites causally associated with DR are predominantly lyso-phospholipids, including 1-stearylglycerolphosphatethanolamine and 1-arachidonicglycerolphosphateinositol, which are both protective factors for DR. Meanwhile, the reverse MR analysis found that both NPDR and PDR can to some extent impact 1-arachidonicglycerolphosphateinositol. The role of sex hormones in diabetic retinopathy is currently uncertain (45). Gangwar et al. (46) found that diabetic patients with hypogonadism exhibited an longer course of diabetes, higher levels of HbA1c and an increased risk of DR. Our study revealed that 4-androsten-3beta,17beta-diol disulfate, an androgenic steroid, can exert a protective effect against any stage of DR.

Additionally, our research has demonstrated the causal relationships between xenobiotics and DR at the genetic level. Erythritol is recommended as a diabetic-safe sweetener due to its metabolic inertness and antioxidant properties and can displayed an endothelium-protective effect in diabetic rats (47). Metabonomic studies have also identified erythritol as a biomarker of diabetes and impaired fasting glucose (11, 48, 49). Piperine is a natural alkaloid from black pepper, and it has been found to protect the retina of diabetic mice by enhancing PEDF expression and suppressing HIF-1/VEGFA pathway (50).

Limited research has been conducted on the role of cofactors and vitamins in DR. Threonate is a metabolic product of vitamin C. A metabolomic analysis showed that the plasma levels of threonate were lower in NPDR patients compared to healthy people (51). The preliminary MR analysis considered threonine as a protective factor, but its meta-analysis result was not significant. Pantothenate, also known as Vitamin B5 or anti-stress vitamin, serves as the precursor of coenzyme A. Wang et al. (52) conducted a comparison of the serum from 15 PDR patients and 15 NPDR patients by untargeted metabolomics and found significant differences in pantothenate levels. Our study further established the bidirectional genetic association between pantothenate and DR.

Our study also identified several potential correlations between DR and nucleotide compounds, such as inosine and guanine. In the purine metabolic cycle, adenosine is initially phosphorylated to adenine, then rapidly deaminated to inosine, and ultimately undergoes a series of reactions to produce nitric oxide (NO) (53). For diabetic patients, NO plays a crucial role in microvascular dysfunction, directly causing lipid and protein peroxidation, tissue damage, ultimately leading to vascular leakage (54). Xia et al. (55) observed the plasma levels of inosine in DR patients were higher than those in the healthy population and diabetic patients without retinopathy. A metabolomics analysis of retina in diabetic mice indicated that, compared to other purine metabolites, adenosine, guanine, and inosine served as excellent biomarkers for predicting DR with higher sensitivity, specificity, and accuracy (56).

Acetylphosphate, as a marker of mitochondrial activity, is considered a metabolic intermediate in the generation of citric acid cycle precursors and often mediates nonenzymatic acetylation (57). Previous studies revealed that acetylation of proteins such as mitofusin 2 (58), histones (59), and P65 (60) contributes to the development of DR. Our study suggested that acetylphosphate may be a risk factor for DR and the acetylation it mediates holds promise as a potential focus for further investigation.

The study identified several metabolic pathways related to DR, some of which have been demonstrated in previous studies (43, 55, 61). As mentioned above, pantothenate and CoA biosynthesis, tryptophan metabolism and purine metabolism may be involved in the biological mechanism for DR (10, 52, 56, 62). In our study, lysine has been identified as a metabolite associated with DR. Given that lysine is involved in the activation of biotin (63), biotin metabolism may play a role in diabetic retinopathy.

The causal correlations between circulating metabolites and DR were assessed for the first time in this MR study. However, there are several restrictions that should be taken into consideration. Firstly, due to the limitations of MR analysis, complete elimination of residual pleiotropy is not achievable, potentially leading to bias. Secondly, the presence of ethnic bias should be noted as the subjects included in both exposure and outcome were of European descent. Hence, caution is warranted when generalizing the findings to other ethnicities. Thirdly, to explore additional blood metabolites associated with DR, we did not correct for *P*-value by false discovery rate correction, which can result in false positives in multiple tests. Fourthly, the direct pathological site of DR is the retina, and the blood-retinal barrier exhibits strict selectivity in the filtration of metabolites (64), indicating the need for further research to analyze changes in metabolites in vitreous and aqueous humor. Finally, it is worth noting that some metabolites in the metabolic profile of this study have unclear structures and functions, which limits our ability to interpret the results of the MR study.

## Conclusion

In conclusion, our study identified 21 circulating metabolites and 4 metabolic pathways associated with DR. These metabolites have the potential to serve as blood biomarkers for DR screening and prevention, as well as potential candidates for further investigation into underlying mechanisms and drug target selection. Both clinical and basic research are required to confirm the roles of these metabolites in DR.

## Data availability statement

The original contributions presented in the study are included in the article/**Supplementary Material**. Further inquiries can be directed to the corresponding author.

## Author contributions

CY: Writing – original draft, Writing – review & editing. YM: Writing – original draft. MY: Writing – original draft. QJ: Writing – review & editing. JX: Writing – review & editing.
