# Supplementary material for: Causal relationships between blood metabolites and diabetic retinopathy: a two-sample Mendelian randomization study
Source: Front Endocrinol (Lausanne). 2024 May 1;15:1383035. doi: 10.3389/fendo.2024.1383035 (PMC11094203; doi:10.3389/fendo.2024.1383035)
Supplement: Supplementary file 2 [file DataSheet_1.docx]

**Causal relationships between genetically determined metabolites and diabetic retinopathy: a two-sample mendelian randomization study**

Chongchao Yang^1, 2^ Yan Ma^1, 2^  Mudi Yao^3^ Qin Jiang^1, 2^ Jinsong Xue^1, 2^*

^1^The Affiliated Eye Hospital, Nanjing Medical University, Nanjing 210029, Jiangsu Province, Chin

^2^The Fourth School of clinical Medicine, Nanjing Medical University, Nanjing 210029, Jiangsu Province, China

^3^ Department of Ophthalmology, The First People's Hospital, Shanghai 200080, China

*** Correspondence:**

Corresponding author: Jinsong Xue

E-mail: 25068411@ qq.com

**Supplementary Material**

1. **Supplementary Tables**

**Supplementary Table 1** List of the identification (ID) for each of the 486 blood metabolites

**Supplementary Table 2** Harmonization data of 486 blood metabolites and All DR

**Supplementary Table 3** Harmonization data of 486 blood metabolites and NPDR

**Supplementary Table 4** Harmonization data of 486 blood metabolites and PDR

**Supplementary Table 5** Causal effects and sensitive analysis of blood metabolites on DR in preliminary MR analysis

**Supplementary Table 6** Causal associations between 486 metabolites and All DR in preliminary MR analysis

**Supplementary Table 7** Causal associations between 486 metabolites and NPDR in preliminary MR analysis

**Supplementary Table 8** Causal associations between 486 metabolites and PDR in preliminary MR analysis

**Supplementary Table 9** Causal effects and sensitive analysis of blood metabolites on DR in replicated MR analysis

**Supplementary Table 10** Causal associations between 486 metabolites and All DR in replicated MR analysis

**Supplementary Table 11** Causal associations between 486 metabolites and NPDR in replicated MR analysis

**Supplementary Table 12** Causal associations between 486 metabolites and PDR in replicated MR analysis

**Supplementary Table 13** Causal effects and sensitive analysis of DR on blood metabolites in reverse MR analysis

**Supplementary Table 14** Metabolic pathways with significant enrichment of blood metabolites
